# Analysis of case-parent trios at a locus with a deletion allele: association of GSTM1 with autism

**DOI:** 10.1186/1471-2156-7-8

**Published:** 2006-02-10

**Authors:** Steven Buyske, Tanishia A Williams, Audrey E Mars, Edward S Stenroos, Sue X Ming, Rong Wang, Madhura Sreenath, Marivic F Factura, Chitra Reddy, George H Lambert, William G Johnson

**Affiliations:** 1 Departments of Statistics and Genetics, 110 Frelinghuysen Rd, Rutgers University, Piscataway, NJ 08854, USA; 2 Department of Neurology, UMDNJ-Robert Wood Johnson Medical School, 675 Hoes Lane, Piscataway, NJ 08854, USA; 3 Department of Pediatrics, UMDNJ-Robert Wood Johnson Medical School, 675 Hoes Lane, Piscataway, NJ 08854, USA; 4 Center for Childhood Neurotoxicology & Exposure Assessment, UMDNJ-Robert Wood Johnson Medical School, 675 Hoes Lane, Piscataway, NJ 08854, USA; 5 Department of Pediatrics, UMDNJ New Jersey Medical School, 90 Bergen Street, Newark, NJ 07103, USA; 6 Department of Neuroscience, UMDNJ New Jersey Medical School, 90 Bergen Street, Newark, NJ 07103, USA

## Abstract

**Background:**

Certain loci on the human genome, such as glutathione S-transferase M1 (GSTM1), do not permit heterozygotes to be reliably determined by commonly used methods. Association of such a locus with a disease is therefore generally tested with a case-control design. When subjects have already been ascertained in a case-parent design however, the question arises as to whether the data can still be used to test disease association at such a locus.

**Results:**

A likelihood ratio test was constructed that can be used with a case-parents design but has somewhat less power than a Pearson's chi-squared test that uses a case-control design. The test is illustrated on a novel dataset showing a genotype relative risk near 2 for the homozygous GSTM1 deletion genotype and autism.

**Conclusion:**

Although the case-control design will remain the mainstay for a locus with a deletion, the likelihood ratio test will be useful for such a locus analyzed as part of a larger case-parent study design. The likelihood ratio test has the advantage that it can incorporate complete and incomplete case-parent trios as well as independent cases and controls. Both analyses support (*p *= 0.046 for the proposed test, *p *= 0.028 for the case-control analysis) an association of the homozygous GSTM1 deletion genotype with autism.

## Background

### Methodology

Despite technological advances, not all loci in the human genome can readily be fully genotyped using current conventional methods. Incomplete sequence information, unknown splice junction, unknown size of the deletion, and a large amount of homology with nearby sequence can all contribute to such a problem. The GSTM1 locus can be considered a model of such a locus. Heterozygotes involving the GSTM1 deletion, or null, allele cannot be detected using standard genotyping methods [1]. In such a case, the investigator can determine genotype only up to homozygous-deletion/not-homozygous-deletion categorization, serving as a reminder that what we label "genotype" in our data analysis is actually an observed phenotype. Studies involving such loci generally use a case-control contingency table analysis with two categories for genotype.

Contemporary research often uses a family-based association study design in examining a number of loci at once. The question naturally arises as to whether case-parent trio DNA be used to advantage over the case-control contingency table analysis at a locus where heterozygotes cannot be reliably distinguished from one of the homozygote genotypes. This note examines a likelihood ratio test built on possible mating types for a case-parent design. Unlike the well-known transmission disequilbrium test for case-parent trios, the test discussed here does require allele frequency estimates and so is susceptible to population stratification and admixture effects much as a case control analysis is. With that proviso, we examine the performance of the proposed test in simulations, and in a new dataset involving the GSTM1 deletion allele and the autism phenotype.

### Autism and the GSTM1 locus

Autism (autistic disorder) is a pervasive developmental disorder with diagnostic criteria based on abnormal social interactions, language abnormalities, and stereotypies evident prior to 36 months of age [2]. Despite its lack of Mendelian transmission, autism is highly genetically determined [3,4].

The vast majority of cases of autism are unrelated to known teratogens but the phenotypic expression of autism may be affected by the interaction of environmental factors with multiple gene loci. There is evidence supporting a role for oxidative stress in autism [5,6]. Oxidative stress could interact with common functional polymorphic variants of genes that protect against oxidative stress and could thus affect brain development during gestation or possibly after gestation, contributing to expression of autism. Glutathione (GSH) is the most important endogenous antioxidant due to its ability to bind electrophilic substrates through its free sulfhydryl group [7] and is the most abundant non-protein thiol, occurring in millimolar concentrations in human tissues [8]. Low plasma total GSH (tGSH) levels, elevated levels of oxidized GSH (GSSG) and low ratios of tGSH to GSSG have been reported in autism [9].

Glutathione-S-transferases (GSTs), are an important class of antioxidant enzymes that catalyze conjugation of GSH to toxic electrophiles. GSTs are abundant, accounting for up to 10% of cellular protein [10]. Some genetic polymorphisms of GSTs are known to affect enzyme function. It is possible that a functional GST polymorphism could contribute to the pathogenesis of autism, an effect that could be potentiated by reduced levels of GSH, one of the substrates of GSTs. GSTs are Phase II enzymes that conjugate GSH to activated toxins, xenobiotics and metabolites including products of Phase I enzymes such as cytochrome P450 oxidases.

Polymorphic alleles of GSTs have been reported to contribute to a number of human diseases. We focused on the GSTM1*0 polymorphism because the variant allele is a complete gene deletion that lacks function of the GSTM1 enzyme. Homozygosity for GSTM1*0 was reportedly associated with an increased risk of prostate cancer in the presence of either the val/val or the ile/val genotypes of the Phase I enzyme CYP1A1 [11]. Homozygosity for GSTM1*0 was associated with increased risk of bladder cancer [12]. GSTM1*0 contributed to risk of hepatocellular cancer in conjunction with environmental factors [13]. GSTM1*0 contributed to breast cancer risk in conjunction with the val/val genotype of the Phase I enzyme, CYP1A1 [14]. GSTM1*0 also contributed to the risk of small cell lung cancer [15] and asthma [16]. GSTM1 is located on 1pl3.3. At least three reports [17-19] show some evidence of genetic linkage of autism to the region; we are not aware of any genetic association studies of autism in this region.

## Methods

### Likelihood ratio test

For a given bi-allelic locus, there are 15 possible triplets of genotypes for the father-mother-child trios [20,21]. The left half of Table 1 shows these triplets, expressed in terms of the number of full alleles each trio-member has. The table also shows the population frequency of each triplet under Hardy-Weinberg equilibrium (HWE) in the parents, as well as the sampling frequencies under the assumptions that each child is a case and that the relative risk of zero copies (one copy) of the full allele for the disorder in question is *r*_0 _(*r*_1_). The right hand side of the table gives the same information when 2 copies of the full allele cannot be distinguished from 1 copy; 1 or 2 copies are denoted P (for present) and 0 copies are denoted D (for deletion). It does not appear to be possible to test for Hardy-Weinberg equilibrium in this situation.

**Table 1:** Population and Sampling Frequencies of Case-Parent Trios. "F, M, C" refers to Father, Mother, Child.

Actual			Observable			
F, M, C Genotype	Population Frequency^*a*^	Case Frequency^*b*^	F, M, C Genotype	Population Frequency^*a*^	Case Frequency^*b*^	Label

2, 2, 2	*p*^4^	*p*^4^/*M*	P, P, P	*p*^2^(3 - 2*p*)	*p*^2^(1 + 2*r*_1_(1 - *p*))/*M*	a
2, 1, 2	*p*^3^(1 - *p*)	*p*^3^(1 - *p*)/*M*				
2, 1, 1	*p*^3^(1 - *p*)	*r*_1_*p*^3^(1 - *p*)/*M*				
1, 2, 2	*p*^3^(1 - *p*)	*p*^3^(1 - *p*)/*M*				
1, 2, 1	*p*^3^(1 - *p*)	*r*_1_*p*^3^(1 - *p*)/*M*				
1, 1, 2	*p*^2^(1 - *p*)^2^	*p*^2^(1 - *p*)^2^/*M*				
1, 1, 1	2*p*^2^(1 - *p*)^2^	2*r*_1_*p*^2^(1 - *p*)^2^/*M*				
1, 1, 0	*p*^2^(1 - *p*)^2^	*r*_0_*p*^2^(1 - *p*)^2^/*M*	P, P, D	*p*^2^(1 - *p*)^2^	*r*_0_*p*^2^(1 - *p*)^2^/*M*	b
2, 0, 1	*p*^2^(1 - *p*)^2^	*r*_1_*p*^2^(1 - *p*)^2^/*M*	P, D, P	*p*(1 - *p*)^2^	*r*_1_*p*(1 - *p*)^2^/*M*	c
1, 0, 1	*p*(1 - *p*)^3^	*r*_1_*p*(1 - *p*)^3^/*M*				
1, 0, 0	*p*(1 - *p*)^3^	*r*_0_*p*(1 - *p*)^3^/*M*	P, D, D	*p*(1 - *p*)^3^	*r*_0_*p*(1 - *p*)^3^/*M*	d
0, 2, 1	*p*^2^(1 - *p*)^2^	*r*_1_*p*^2^(1 - *p*)^2^/*M*	D, P, P	*p*(1 - *p*)^2^	*r*_1_*p*(1 - *p*)^2^/*M*	e
0, 1, 1	*p*(1 - *p*)^3^	*r*_1_*p*(1 - *p*)^3^/*M*				
0, 1, 0	*p*(1 - *p*)^3^	*r*_0_*p*(1 - *p*)^3^/*M*	D, P, D	*p*(1 - *p*)^3^	*r*_0_*p*(1 - *p*)^3^/*M*	f
0, 0, 0	(1 - *p*)^4^	*r*_0_(1 - *p*)^4^/*M*	D, D, D	(1 - *p*)^4^	*r*_0_(1 - *p*)^4^/*M*	g

Note: *M *= *p*^2 ^+ 2*r*_1_*p*(1 - *p*) + *r*_0_(1 - *p*)^2^. The allele frequency of the full allele is denoted by *p*.

^*a *^Under the assumption of Hardy-Weinberg equilibrium.

^*b *^Under the assumption of Hardy-Weinberg equilibrium, and a risk of *r*_0 _(*r*_1_) for a child with zero copies (one copy) of full allele relative to the risk to a child with two copies of the full allele.

Case-parent trios can be categorized into one of the 7 types on the right of the table. The resulting counts will follow a multinomial distribution with cell probabilities as given in the table. One can then construct a likelihood under a model with

1. *r*_1 _= *r*_0 _= 1,

2. *r*_1 _= 1 but *r*_0 _unconstrained, or

3. *r*_0 _and *r*_1 _both unconstrained.

Model 2 might correspond to a scientific hypothesis that either one or two copies of the full allele provides the same biological functionality, while model 3 might correspond biologically to a dose-response model (although *r*_1 _is not constrained to lie between 1 and *r*_0_). Other models, such as *r*_1 _= r0
 MathType@MTEF@5@5@+=feaafiart1ev1aaatCvAUfKttLearuWrP9MDH5MBPbIqV92AaeXatLxBI9gBaebbnrfifHhDYfgasaacH8akY=wiFfYdH8Gipec8Eeeu0xXdbba9frFj0=OqFfea0dXdd9vqai=hGuQ8kuc9pgc9s8qqaq=dirpe0xb9q8qiLsFr0=vr0=vr0dc8meaabaqaciaacaGaaeqabaqabeGadaaakeaadaGcaaqaaiabdkhaYnaaBaaaleaacqaIWaamaeqaaaqabaaaaa@2F43@ or *r*_1 _= *r*_0 _are also possible. The likelihood ratio test has a test statistic equal to twice the difference in the maximized log-likelihoods of the relevant models. Asymptotically that test statistic is distributed as a chi-squared random variable with degrees of freedom equal to the number of additional parameters estimated, namely 1 for the second model versus the first or the third versus the second, and two for the third versus the first. In all models, *p*, the frequency of the full allele, will be estimated.

Under Model 2, the maximum likelihood estimator of *r*_0 _is simply

r^0=nm1−q^2q^2,
 MathType@MTEF@5@5@+=feaafiart1ev1aaatCvAUfKttLearuWrP9MDH5MBPbIqV92AaeXatLxBI9gBaebbnrfifHhDYfgasaacH8akY=wiFfYdH8Gipec8Eeeu0xXdbba9frFj0=OqFfea0dXdd9vqai=hGuQ8kuc9pgc9s8qqaq=dirpe0xb9q8qiLsFr0=vr0=vr0dc8meaabaqaciaacaGaaeqabaqabeGadaaakeaacuWGYbGCgaqcamaaBaaaleaacqaIWaamaeqaaOGaeyypa0ZaaSaaaeaacqWGUbGBaeaacqWGTbqBaaWaaSaaaeaacqaIXaqmcqGHsislcuWGXbqCgaqcamaaCaaaleqabaGaeGOmaidaaaGcbaGafmyCaeNbaKaadaahaaWcbeqaaiabikdaYaaaaaGccqGGSaalaaa@3B40@

where *m *is the total number of cases with the full allele present, *n *is the total number of cases homozygous for the null allele, and q^
 MathType@MTEF@5@5@+=feaafiart1ev1aaatCvAUfKttLearuWrP9MDH5MBPbIqV92AaeXatLxBI9gBaebbnrfifHhDYfgasaacH8akY=wiFfYdH8Gipec8Eeeu0xXdbba9frFj0=OqFfea0dXdd9vqai=hGuQ8kuc9pgc9s8qqaq=dirpe0xb9q8qiLsFr0=vr0=vr0dc8meaabaqaciaacaGaaeqabaqabeGadaaakeaacuWGXbqCgaqcaaaa@2E27@ = 1 - p^
 MathType@MTEF@5@5@+=feaafiart1ev1aaatCvAUfKttLearuWrP9MDH5MBPbIqV92AaeXatLxBI9gBaebbnrfifHhDYfgasaacH8akY=wiFfYdH8Gipec8Eeeu0xXdbba9frFj0=OqFfea0dXdd9vqai=hGuQ8kuc9pgc9s8qqaq=dirpe0xb9q8qiLsFr0=vr0=vr0dc8meaabaqaciaacaGaaeqabaqabeGadaaakeaacuWGWbaCgaqcaaaa@2E25@ is the estimated frequency of the null allele. The estimator r^0
 MathType@MTEF@5@5@+=feaafiart1ev1aaatCvAUfKttLearuWrP9MDH5MBPbIqV92AaeXatLxBI9gBaebbnrfifHhDYfgasaacH8akY=wiFfYdH8Gipec8Eeeu0xXdbba9frFj0=OqFfea0dXdd9vqai=hGuQ8kuc9pgc9s8qqaq=dirpe0xb9q8qiLsFr0=vr0=vr0dc8meaabaqaciaacaGaaeqabaqabeGadaaakeaacuWGYbGCgaqcamaaBaaaleaacqaIWaamaeqaaaaa@2F43@ is thus simply the observed ratio of the two detectable genotypes among the cases divided by the ratio expected under the null hypothesis.

When both *r*_0 _and *r*_1 _are estimated, the maximum likelihood estimators are

r^0=np^2q^(a−mp^)
 MathType@MTEF@5@5@+=feaafiart1ev1aaatCvAUfKttLearuWrP9MDH5MBPbIqV92AaeXatLxBI9gBaebbnrfifHhDYfgasaacH8akY=wiFfYdH8Gipec8Eeeu0xXdbba9frFj0=OqFfea0dXdd9vqai=hGuQ8kuc9pgc9s8qqaq=dirpe0xb9q8qiLsFr0=vr0=vr0dc8meaabaqaciaacaGaaeqabaqabeGadaaakeaacuWGYbGCgaqcamaaBaaaleaacqaIWaamaeqaaOGaeyypa0ZaaSaaaeaacqWGUbGBcuWGWbaCgaqcamaaCaaaleqabaGaeGOmaidaaaGcbaGafmyCaeNbaKaacqGGOaakcqWGHbqycqGHsislcqWGTbqBcuWGWbaCgaqcaiabcMcaPaaaaaa@3CAB@

and

r^1=(m−a)p^2q^(a−mp^),
 MathType@MTEF@5@5@+=feaafiart1ev1aaatCvAUfKttLearuWrP9MDH5MBPbIqV92AaeXatLxBI9gBaebbnrfifHhDYfgasaacH8akY=wiFfYdH8Gipec8Eeeu0xXdbba9frFj0=OqFfea0dXdd9vqai=hGuQ8kuc9pgc9s8qqaq=dirpe0xb9q8qiLsFr0=vr0=vr0dc8meaabaqaciaacaGaaeqabaqabeGadaaakeaacuWGYbGCgaqcamaaBaaaleaacqaIXaqmaeqaaOGaeyypa0ZaaSaaaeaacqGGOaakcqWGTbqBcqGHsislcqWGHbqycqGGPaqkcuWGWbaCgaqcaaqaaiabikdaYiqbdghaXzaajaGaeiikaGIaemyyaeMaeyOeI0IaemyBa0MafmiCaaNbaKaacqGGPaqkaaGaeiilaWcaaa@413E@

with *m*, *n*, p^
 MathType@MTEF@5@5@+=feaafiart1ev1aaatCvAUfKttLearuWrP9MDH5MBPbIqV92AaeXatLxBI9gBaebbnrfifHhDYfgasaacH8akY=wiFfYdH8Gipec8Eeeu0xXdbba9frFj0=OqFfea0dXdd9vqai=hGuQ8kuc9pgc9s8qqaq=dirpe0xb9q8qiLsFr0=vr0=vr0dc8meaabaqaciaacaGaaeqabaqabeGadaaakeaacuWGWbaCgaqcaaaa@2E25@ as before and *a *the number of (*P*, *P*, *P*) trios. These estimates do not admit a simple description as when only *r*_0 _is estimated.

For all three models, *p *can be estimated as the solution to a quadratic or cubic equation, although in case (3) there is a particularly simple form of p^
 MathType@MTEF@5@5@+=feaafiart1ev1aaatCvAUfKttLearuWrP9MDH5MBPbIqV92AaeXatLxBI9gBaebbnrfifHhDYfgasaacH8akY=wiFfYdH8Gipec8Eeeu0xXdbba9frFj0=OqFfea0dXdd9vqai=hGuQ8kuc9pgc9s8qqaq=dirpe0xb9q8qiLsFr0=vr0=vr0dc8meaabaqaciaacaGaaeqabaqabeGadaaakeaacuWGWbaCgaqcaaaa@2E25@ = (2*b *+ *d *+ *f*)/(2*n*), where *b*, *d*, and *f *are as in the mating type table and represent the counts in cells with non-obligate null homozygous cases.

The discussion above applies when the data consists only of completely case-parent trios, but the test can easily accommodate data on cases with a single genotyped parent, cases with no parental genotypes, and controls. Control subjects, in particular, will yield more accurate allele frequency estimates. With the additional subject types, the likelihood factors into a complete trio term, an incomplete trio term, a case-only term, and a control-only term. For cases with incomplete parental genotyping, the cells in Table 1 are simply collapsed over the parent's unknown genotypes. For example, when the mother's genotype is unknown, the probability of the father and child both having the "Present" allele is simply the sum of the probabilities of the (P, P, P) and (P, D, P) types (cells labeled a and c in the table). With the greater variety of data types, the maximum likelihood estimators no longer have closed forms. The likelihood, however, remains straightforward. Under each model and for each data type, the probability of an observation belonging to a particular cell is a function of *p*, *r*_0_, and *r*_1_. The overall likelihood is a product of the likelihoods for each data type. The likelihood can then be maximized using standard numerical techniques. Code for the R statistical environment [22] containing functions for calculating test statistics, estimates, and confidence intervals is available [see Additional file 1].

### Autism study

The cohort (70 nuclear families) for the autism association study was ascertained through the New Jersey Center for Outreach and Services for the Autism Community (COSAC) and the UMDNJ-RWJMS Department of Pediatrics-Division of Neurodevelopmental Disabilities. Each affected individual had diagnosis by the Autism Diagnostic Interview-Revised (ADI-R) and the Autism Diagnostic Observation Schedule-Generic (ADOS-G) [23,24]. Blood samples were drawn from members of autism families and from unrelated, unaffected controls ascertained from UMDNJ clinics and individuals married into dominant pedigrees of other disorders. This study was approved by the Institutional Review Board of UMDNJ-Robert Wood Johnson Medical School and UMDNJ-New Jersey Medical School.

Genotyping of the GSTM1*0 whole gene deletion polymorphism was carried out by the method of Yang et al. [25] with specific primers using a PCR method with the beta-globin gene amplified as a positive control for PCR efficiency. PCR products were separated on polyacrylamide gels and visualized with ethidium bromide. The GSTM1 product was about 200 bp and the beta-globin product was about 250 bp. In the presence of a positive betaglobin band, the absence of the GSTM1 band was interpreted as homozygosity for the whole gene deletion allele [25].

## Results

### Simulations

To study the power of the likelihood ratio tests compared to the usual case control contingency table analysis, we performed a number of simulations, the results of which are shown in Tables 2 and 3. Each cell in the tables represents 10,000 runs. The simulations vary the deletion allele frequency *q *(so the observed homozygous deletion genotype frequency is *q*^2^), the relative risks *r*_0 _and *r*_1 _for zero or one copies of the full allele as compared to the risk for the genotype homozygous for the full allele, and the number of trios (either 50 or 200). All simulations use a prevalence of 0.001 for the disorder. For the case control simulations there were twice the number of controls as cases, so that each test involved the same amount of genotyping. The test statistic for the case control simulations was the Pearson chi-squared statistic without continuity correction. Other contingency table test statistics give very similar results (data not shown). Controls were not used for the likelihood ratio tests in these Tables 2 and 3, but Table 4 shows a selection of results when the controls are used in the likelihood ratio tests. The table shows the case just for *r*_0 _= 2, but the general pattern holds for other values of *r*_0 _(results not shown). Table 4 illustrates that the 1-df likelihood ratio test utilizing the controls data has slightly more power than the contingency table analysis under a recessive model and slightly less power under the multiplicative model. Of course, using 2 controls for each case represents 5/3 as much genotyping for the likelihood ratio tests as for the contingency table analyses. The table therefore also includes the power when the case:control ratio is 1:4, so that the total genotyping is the same as for the likelihood ratio tests. Not surprisingly, this design generally has greatest power except under the dominant genetic model.

**Table 2:** Power with 50 Case-Parent Trios and 100 Controls. CC = Pearson's Chi-Squared Test, LR 1 = likelihood ratio test with 1 df, LR 2 = likelihood ratio test with 2 df. Controls were used only for the Chi-Squared test.

		*r*_1 _= 1			*r*_1 _= r0 MathType@MTEF@5@5@+=feaafiart1ev1aaatCvAUfKttLearuWrP9MDH5MBPbIqV92AaeXatLxBI9gBaebbnrfifHhDYfgasaacH8akY=wiFfYdH8Gipec8Eeeu0xXdbba9frFj0=OqFfea0dXdd9vqai=hGuQ8kuc9pgc9s8qqaq=dirpe0xb9q8qiLsFr0=vr0=vr0dc8meaabaqaciaacaGaaeqabaqabeGadaaakeaadaGcaaqaaiabdkhaYnaaBaaaleaacqaIWaamaeqaaaqabaaaaa@2F43@			*r*_1 _= *r*_0_		
	*r*_0_	CC	LR 1	LR 2	CC	LR 1	LR 2	CC	LR 1	LR 2
*q *= .25	1.0	0.04	0.06	0.06						
	1.5	0.11	0.10	0.09	0.08	0.07	0.07	0.06	0.07	0.07
	2.0	0.23	0.20	0.19	0.15	0.09	0.09	0.10	0.07	0.08
	2.5	0.38	0.32	0.34	0.23	0.12	0.14	0.12	0.07	0.09
	3.0	0.53	0.46	0.47	0.32	0.17	0.18	0.14	0.07	0.10
*q *= .50	1.0	0.05	0.05	0.05						
	1.5	0.20	0.16	0.14	0.11	0.08	0.07	0.06	0.06	0.07
	2.0	0.47	0.38	0.31	0.23	0.15	0.12	0.08	0.06	0.07
	2.5	0.72	0.62	0.52	0.36	0.22	0.18	0.09	0.07	0.09
	3.0	0.86	0.79	0.70	0.49	0.29	0.25	0.10	0.07	0.10
*q *= .75	1.0	0.05	0.05	0.05						
	1.5	0.21	0.17	0.14	0.11	0.09	0.08	0.06	0.06	0.06
	2.0	0.47	0.39	0.31	0.20	0.15	0.12	0.06	0.06	0.07
	2.5	0.68	0.59	0.48	0.30	0.22	0.18	0.06	0.06	0.08
	3.0	0.82	0.72	0.62	0.39	0.28	0.23	0.07	0.06	0.08

**Table 3:** Power with 200 Case-Parent Trios and 400 Controls. CC = Pearson's Chi-Squared Test, LR 1 = likelihood ratio test with 1 df, LR 2 = likelihood ratio test with 2 df. Controls were used only for the Chi-Squared test.

		*r*_1 _= 1			*r*_1 _= r0 MathType@MTEF@5@5@+=feaafiart1ev1aaatCvAUfKttLearuWrP9MDH5MBPbIqV92AaeXatLxBI9gBaebbnrfifHhDYfgasaacH8akY=wiFfYdH8Gipec8Eeeu0xXdbba9frFj0=OqFfea0dXdd9vqai=hGuQ8kuc9pgc9s8qqaq=dirpe0xb9q8qiLsFr0=vr0=vr0dc8meaabaqaciaacaGaaeqabaqabeGadaaakeaadaGcaaqaaiabdkhaYnaaBaaaleaacqaIWaamaeqaaaqabaaaaa@2F43@			*r*_1 _= *r*_0_		
	*r*_0_	CC	LR 1	LR 2	CC	LR 1	LR 2	CC	LR 1	LR 2
*q *= .25	1.0	0.05	0.05	0.05						
	1.5	0.26	0.22	0.17	0.18	0.09	0.08	0.11	0.05	0.07
	2.0	0.64	0.57	0.47	0.43	0.19	0.16	0.20	0.06	0.09
	2.5	0.88	0.83	0.80	0.65	0.32	0.28	0.31	0.06	0.12
	3.0	0.97	0.95	0.94	0.82	0.46	0.41	0.39	0.06	0.15
*q *= .50	1.0	0.05	0.05	0.05						
	1.5	0.58	0.49	0.39	0.29	0.16	0.13	0.10	0.06	0.09
	2.0	0.96	0.92	0.86	0.66	0.39	0.34	0.16	0.06	0.14
	2.5	1.00	0.99	0.99	0.88	0.63	0.57	0.22	0.07	0.19
	3.0	1.00	1.00	1.00	0.96	0.79	0.75	0.26	0.06	0.24
*q *= .75	1.0	0.05	0.05	0.05						
	1.5	0.62	0.51	0.41	0.25	0.17	0.14	0.06	0.06	0.07
	2.0	0.97	0.92	0.86	0.59	0.41	0.35	0.07	0.06	0.11
	2.5	1.00	0.99	0.98	0.81	0.63	0.55	0.08	0.06	0.12
	3.0	1.00	1.00	1.00	0.92	0.79	0.71	0.09	0.06	0.15

**Table 4:** Power Using Controls in All Tests. CC^*a *^= Pearson's Chi-Square with twice as many controls as cases. CC^*b *^= Pearson's Chi-Square with four times as many controls as cases. LR 1 = likelihood ratio test with 1 df, LR 2 = likelihood ratio test with 2 df. Both likelihood tests used twice as many controls as cases.

		*r*_1 _= 1		*r*_1 _= r0 MathType@MTEF@5@5@+=feaafiart1ev1aaatCvAUfKttLearuWrP9MDH5MBPbIqV92AaeXatLxBI9gBaebbnrfifHhDYfgasaacH8akY=wiFfYdH8Gipec8Eeeu0xXdbba9frFj0=OqFfea0dXdd9vqai=hGuQ8kuc9pgc9s8qqaq=dirpe0xb9q8qiLsFr0=vr0=vr0dc8meaabaqaciaacaGaaeqabaqabeGadaaakeaadaGcaaqaaiabdkhaYnaaBaaaleaacqaIWaamaeqaaaqabaaaaa@2F43@		*r*_1 _= *r*_0_	
		LR 1	LR 2	LR 1	LR 2	LR 1	LR 2
*q*	*r*_0_	CC^*a*^	CC^*b*^	CC^*a*^	CC^*b*^	CC^*a*^	CC^*b*^

50 Case Trios							
.25	2.0	0.24	0.20	0.13	0.13	0.07	0.11
		0.23	0.30	0.15	0.20	0.09	0.13
.50	2.0	0.51	0.42	0.21	0.19	0.06	0.11
		0.46	0.55	0.23	0.28	0.08	0.09
.75	2.0	0.53	0.43	0.20	0.16	0.06	0.08
		0.48	0.54	0.19	0.22	0.06	0.06
200 Case Trios							
.25	2.0	0.69	0.60	0.35	0.33	0.11	0.27
		0.64	0.72	0.42	0.50	0.21	0.25
.50	2.0	0.98	0.96	0.64	0.58	0.09	0.27
		0.97	0.98	0.66	0.75	0.16	0.18
.75	2.0	0.98	0.96	0.61	0.53	0.06	0.13
		0.97	0.99	0.59	0.66	0.07	0.07

To examine the effect of incomplete parental genotypes, we also performed power analyses with some complete case-parent trios replaced with trios with only one parent genotyped (data not shown). When the number of subjects genotyped was held constant (i.e., *n *completely genotyped trios replaced with 3*n*/2 one-parent-genotyped trios), the power differed by only a few percentage points. This result indicates that a case-parent trio with one parent genotyped carries roughly 2/3 of the information of a complete trio. It may well be the case that methods that can distinguish heterozygotes from both homozgyotes are available, but are more expensive than methods that give partial information. To examine how much information is lost by partial genotyping, we calculated the relative efficiency, in terms of sample sizes, of using partial genotyping versus fully-informative genotyping. Table 5 shows the percentage relative efficiency for recessive and multiplicative genetic models. We do not show the comparison for the dominant model, as the power performance of the proposed test with partial genotyping is so poor. The first pair of columns show the efficiency of the 1-df proposed test with partial genotyping compared to the TDT test with fully informative genotyping. The TDT is known to perform poorly under a recessive generating model, so the second pair of columns compares the 1-df proposed test with Schaid's 2-df likelihood ratio test with full genotyping [26]. Schaid's test is robust across many genetic models. The last pair of columns shows Schaid's 2-df test compared with the proposed 2-df test. The power of the TDT and Schaid's test was calculated using Knapp's and Schaid's methods [26,27] and compared with the results in Table 3.

**Table 5:** Relative Efficiency of Partial Genotyping versus Fully Informative Genotyping. The table gives the percentage relative efficiency of partial (homozygous deletion versus other) genotyping with the proposed test compared to full genotyping. Numbers less than 100% indicate the proposed test with partial genotyping is less efficient than the standard test with full genotyping. LR 1 and LR 2 refer to the proposed likelihood ratio tests with 1- and 2-df, respectively. TDT refers to the transmission-disequilibrium test. Schaid *LR*_*Gen *_refers to Schaid's 2-df likelihood ratio test [26]. Calculations are based on Table 3.

		LR 1 versus		LR 1 versus		LR 2 versus	
		TDT		Schaid *LR*_*Gen*_			
*q*	*r*_0_	*r*_1 _= 1	*r*_1 _= r0 MathType@MTEF@5@5@+=feaafiart1ev1aaatCvAUfKttLearuWrP9MDH5MBPbIqV92AaeXatLxBI9gBaebbnrfifHhDYfgasaacH8akY=wiFfYdH8Gipec8Eeeu0xXdbba9frFj0=OqFfea0dXdd9vqai=hGuQ8kuc9pgc9s8qqaq=dirpe0xb9q8qiLsFr0=vr0=vr0dc8meaabaqaciaacaGaaeqabaqabeGadaaakeaadaGcaaqaaiabdkhaYnaaBaaaleaacqaIWaamaeqaaaqabaaaaa@2F43@	*r*_1 _= 1	*r*_1 _= r0 MathType@MTEF@5@5@+=feaafiart1ev1aaatCvAUfKttLearuWrP9MDH5MBPbIqV92AaeXatLxBI9gBaebbnrfifHhDYfgasaacH8akY=wiFfYdH8Gipec8Eeeu0xXdbba9frFj0=OqFfea0dXdd9vqai=hGuQ8kuc9pgc9s8qqaq=dirpe0xb9q8qiLsFr0=vr0=vr0dc8meaabaqaciaacaGaaeqabaqabeGadaaakeaadaGcaaqaaiabdkhaYnaaBaaaleaacqaIWaamaeqaaaqabaaaaa@2F43@	*r*_1 _= 1	*r*_1 _= r0 MathType@MTEF@5@5@+=feaafiart1ev1aaatCvAUfKttLearuWrP9MDH5MBPbIqV92AaeXatLxBI9gBaebbnrfifHhDYfgasaacH8akY=wiFfYdH8Gipec8Eeeu0xXdbba9frFj0=OqFfea0dXdd9vqai=hGuQ8kuc9pgc9s8qqaq=dirpe0xb9q8qiLsFr0=vr0=vr0dc8meaabaqaciaacaGaaeqabaqabeGadaaakeaadaGcaaqaaiabdkhaYnaaBaaaleaacqaIWaamaeqaaaqabaaaaa@2F43@

.25	1.5	240	24	120	34	70	14
	2.5	218	27	107	35	98	29
.50	1.5	148	44	119	62	90	48
	2.5	138	51	107	64	93	56
.75	1.5	114	76	121	105	94	82
	2.5	108	77	112	97	95	77

### Autism and the GSTM1 deletion allele

The allele frequencies of GSTM1 are known to vary with the population. For this analysis, the study sample was restricted to the largest racial and ethnic group, namely those self-identifying as Non-Hispanic White. The published homozygous deletion genotype frequency in this population is about 0.5 [28], suggesting a deletion allele frequency *q *of about 0.7. The final sample reported here consists of 54 complete case-parent trios and 172 controls. Of the cases, 45 were diagnosed with autistic disorder on both the ADI-R and ADOS-G, while 9 were diagnosed with pervasive developmental disorder not otherwise specified on one instrument but autistic disorder on the other.

The observed genotypes are shown in Table 6. The chi-squared test statistics are 4.83 for Pearson's, 3.98 for the 1-df LRT, and 3.98 for the 2-df LRT (based on the next section, the 2-df LRT would not be recommended in this situation, but is included here for completeness), giving *p*-values of 0.028, 0.046, and 0.137, respectively, with controls included in all tests. The genotype relative risk estimates are r^0
 MathType@MTEF@5@5@+=feaafiart1ev1aaatCvAUfKttLearuWrP9MDH5MBPbIqV92AaeXatLxBI9gBaebbnrfifHhDYfgasaacH8akY=wiFfYdH8Gipec8Eeeu0xXdbba9frFj0=OqFfea0dXdd9vqai=hGuQ8kuc9pgc9s8qqaq=dirpe0xb9q8qiLsFr0=vr0=vr0dc8meaabaqaciaacaGaaeqabaqabeGadaaakeaacuWGYbGCgaqcamaaBaaaleaacqaIWaamaeqaaaaa@2F43@ = 1.85 for the 1-df test, r^0
 MathType@MTEF@5@5@+=feaafiart1ev1aaatCvAUfKttLearuWrP9MDH5MBPbIqV92AaeXatLxBI9gBaebbnrfifHhDYfgasaacH8akY=wiFfYdH8Gipec8Eeeu0xXdbba9frFj0=OqFfea0dXdd9vqai=hGuQ8kuc9pgc9s8qqaq=dirpe0xb9q8qiLsFr0=vr0=vr0dc8meaabaqaciaacaGaaeqabaqabeGadaaakeaacuWGYbGCgaqcamaaBaaaleaacqaIWaamaeqaaaaa@2F43@ = 1.76 and r^1
 MathType@MTEF@5@5@+=feaafiart1ev1aaatCvAUfKttLearuWrP9MDH5MBPbIqV92AaeXatLxBI9gBaebbnrfifHhDYfgasaacH8akY=wiFfYdH8Gipec8Eeeu0xXdbba9frFj0=OqFfea0dXdd9vqai=hGuQ8kuc9pgc9s8qqaq=dirpe0xb9q8qiLsFr0=vr0=vr0dc8meaabaqaciaacaGaaeqabaqabeGadaaakeaacuWGYbGCgaqcamaaBaaaleaacqaIXaqmaeqaaaaa@2F45@ = 0.94 for the 2-df test. Estimates of *q *are 0.73 under model (1) and 0.71 under both models (2) and (3). When controls were not used in the likelihood ratio tests, the chi-squared values were 0.80 and 1.31 for the 1- and 2-df tests, respectively, giving *p*-values of 0.371 and 0.521. The results for the case-control analysis and the 1-df likelihood ratio test (utilizing controls) are repeated in Table 7.

**Table 6:** Observed Genotypes in GSTM1 and Autism Association Study.

Mating Type	Count
Parent-Case Trios:	
P P P	8
P P D	2
P D P	6
P D D	4
D P P	5
D P D	8
D D D	21
Controls:	
P	90
D	82

**Table 7:** Results for GSTM1 and Autism Association Study. "Pearson" refers to Pearson's chi-square analysis of the case-control data. "Likelihood Ratio Test" refers to the 1-df test discussed in the text, in this case using the full information in Table 6. OR = Odds Ratio, RR = Relative Risk of homozygous deletion genotype relative (*r*_1 _in the text).

Method	Pearson	Likelihood Ratio Test
Chi-square	4.84	3.97
p	0.028	0.046
Effect Size (95% Conffidence Interval)	OR = 2.02 (1.03; 4.04)	RR = 1.85 (1.07; 3.30)
Null allele frequency	0.69	0.71

## Discussion

### Proposed test

The simulations show that the 1-df likelihood ratio test has somewhat less power than the case control approach under a recessive genetic model (*r*_1 _= 1) and much less power under an multiplicative model (*r*_1 _= r0
 MathType@MTEF@5@5@+=feaafiart1ev1aaatCvAUfKttLearuWrP9MDH5MBPbIqV92AaeXatLxBI9gBaebbnrfifHhDYfgasaacH8akY=wiFfYdH8Gipec8Eeeu0xXdbba9frFj0=OqFfea0dXdd9vqai=hGuQ8kuc9pgc9s8qqaq=dirpe0xb9q8qiLsFr0=vr0=vr0dc8meaabaqaciaacaGaaeqabaqabeGadaaakeaadaGcaaqaaiabdkhaYnaaBaaaleaacqaIWaamaeqaaaqabaaaaa@2F43@). None of the tests performed well under a dominant model (*r*_1 _= *r*_0_), but with a deletion allele, likely to result in a loss of function, this model seems less likely on biological grounds. It could, however, arise when partial loss of function reduces the gene product below a functional threshold. The 2-df likelihood ratio test was slightly less powerful than the 1-df test for the multiplicative model and considerably more powerful under a dominant model. It is much less powerful than the 1-df test under the recessive model, which, of course, is the genetic model for which the 1-df test model is correct. All of the tests have low power under a dominant model. If this situation is suspected one the expensive of fully informative genotyping followed by a standard test may be worthwhile. If the use of the proposed tests can be avoided when biology suggests a dominant risk model holds, the 2-df test does not appear to hold any power advantage over the 1-df test.

An advantage of a likelihood-based test is that variants can easily be incorporated. Data from complete case-parent trios, incomplete trios, individual cases, and controls can all be used in the tests described here. If full genotypes distinguishing heterzygotes were available on some study participants the likelihood could be modified to incorporate them. The likelihood can also be modified for testing specific genetic models. One could even potentially incorporate parent-of-origin effects as has been done by Weinberg *et al*. for fully genotyped trios [21].

An important weakness of the proposed test is its reliance on Hardy-Weinberg equilibrium among parents. The test is designed for the situation where heterozygotes cannot be distinguished from one of the homozygotes, a situation where Hardy-Weinberg equilibrium cannot be tested. It does not appear that this weakness can be overcome by statistical methods. However, the most common causes of the failure of HWE is likely to be genotyping error or population stratification. The case-control method is vulnerable to these effects as well, so this weakness of the proposed test is no worse than that of the existing method.

### Autism and the GSTM1 deletion allele

The full data available, namely case-parent trios along with controls, gives evidence of a heightened risk for autism for GSTM1*0 homozygotes. The population frequency of that genotype is large, but the genotype is presumably interacting with other genetic and environmental risk factors. Absence of the GSTM1 gene in GSTM1*0 homozygotes could lead to failure of individuals with autism to detoxify important compounds, including some that could be agents or products of oxidative stress.

Further studies are needed to confirm these observations. The present findings could be consistent with the hypothesis of a gene-environment interaction that alters the expression of autism because GSTs are detoxification enzymes that conjugate absorbed xenobiotics. These findings could lead to documentation and identification of an exogenous or endogenous moiety interacting with GSTs to contribute to autism and a mechanism of action of select environmental chemicals in contributing to the phenotypic presentation of autism.

## Conclusion

As researchers increasingly study larger sets of candidate loci at a time, they will occasionally find that their study design may not be best for a specific locus. While a case-parent design offers many advantages at most loci, it has not generally been considered possible to use such a design to test a locus where the heterozygote cannot be reliably detected. We have demonstrated that, with the risk of the additional assumption of Hardy-Weinberg equilibrium, it is possible to construct such a test. For the same number of genotyped subjects, the resulting test has less power than a Pearson's chi-squared test using cases and controls. If controls can be added, the proposed test has slightly more power, but at a cost of additional genotyping; if that genotyping were instead dedicated to additional controls, the case-control analysis would maintain its superiority in power. The 2-df test appears to be most useful only when a dominant model for the deletion allele is suspected, but would require a large sample in that circumstance. The 1-df test, however, is more generally worthwhile when the study participants have already been assembled. It has the advantage that it can be used with complete and incomplete trios as well as independent cases and controls With respect to the association study of the GSTM1 locus with autism, both the traditional case-control analysis and the 1-df likelihood ratio test (utilizing controls) support (at *p *= 0.028 and *p *= 0.046, respectively) the association of the homozygous GSTM1 deletion genotype with an increased risk of autism. There is no evidence that the heterozygous genotype contributes to any increased risk.

## Authors' contributions

SB developed the statistical method, performed the data analysis, and drafted the manuscript. TAW assisted with accrual, performed molecular genetic work, managed the data, and helped to draft the manuscript. AEM diagnosed cases and assisted with accrual. ESS performed molecular genetic work and data management. SXM, MFF, CR, and GHL assisted with accrual. RW and MS performed molecular genetic work. WGJ conceived and designed the GSTM1 and autism study and helped to draft the manuscript.

## Supplementary Material

Additional File 1 This text file contains the complete R code needed for performing the test described in this paper, as well as a utility function for counting the case-parent types. Click here for file
